# Hormonal and non-hormonal oral contraceptives given long-term to pubertal rats differently affect bone mass, quality and metabolism

**DOI:** 10.3389/fendo.2023.1233613

**Published:** 2023-08-17

**Authors:** Konica Porwal, Shivani Sharma, Saroj Kumar, Manendra Singh Tomar, Sreyanko Sadhukhan, Swati Rajput, Chirag Kulkarni, Ashutosh Shrivastava, Navin Kumar, Naibedya Chattopadhyay

**Affiliations:** ^1^ Division of Endocrinology and Centre for Research in Anabolic Skeletal Targets in Health and Illness (ASTHI), Council of Scientific & Industrial Research-Central Drug Research Institute, Lucknow, India; ^2^ Academy of Scientifc and Innovative Research (AcSIR), Ghaziabad, India; ^3^ Department of Mechanical Engineering, Indian Institute of Technology Ropar, Rupnagar, Punjab, India; ^4^ Faculty of Medicine, King George’s Medical University, Lucknow, India

**Keywords:** oral contraceptives, bone strength, bone composition, mineralization, bone metabolism

## Abstract

**Introduction:**

We investigated the effects of hormonal and non-hormonal oral contraceptives (OCs) on bone mass, mineralization, composition, mechanical properties, and metabolites in pubertal female SD rats.

**Methods:**

OCs were given for 3-, and 7 months at human equivalent doses. The combined hormonal contraceptive (CHC) was ethinyl estradiol and progestin, whereas the non-hormonal contraceptive (NHC) was ormeloxifene. MicroCT was used to assess bone microarchitecture and BMD. Bone formation and mineralization were assessed by static and dynamic histomorphometry. The 3-point bending test, nanoindentation, FTIR, and cyclic reference point indentation (cRPI) measured the changes in bone strength and material composition. Bone and serum metabolomes were studied to identify potential biomarkers of drug efficacy and safety and gain insight into the underlying mechanisms of action of the OCs.

**Results:**

NHC increased bone mass in the femur metaphysis after 3 months, but the gain was lost after 7 months. After 7 months, both OCs decreased bone mass and deteriorated trabecular microarchitecture in the femur metaphysis and lumbar spine. Also, both OCs decreased the mineral: matrix ratio and increased the unmineralized matrix after 7 months. After 3 months, the OCs increased carbonate: phosphate and carbonate: amide I ratios, indicating a disordered hydroxyapatite crystal structure susceptible to resorption, but these changes mostly reversed after 7 months, indicating that the early changes contributed to demineralization at the later time. In the femur 3-point bending test, CHC reduced energy storage, resilience, and ultimate stress, indicating increased susceptibility to micro-damage and fracture, while NHC only decreased energy storage. In the cyclic loading test, both OCs decreased creep indentation distance, but CHC increased the average unloading slope, implying decreased microdamage risk and improved deformation resistance by the OCs. Thus, reduced bone mineralization by the OCs appears to affect bone mechanical properties under static loading, but not its cyclic loading ability. When compared to an age-matched control, after 7 months, CHC affected 24 metabolic pathways in bone and 9 in serum, whereas NHC altered 17 in bone and none in serum. 6 metabolites were common between the serum and bone of CHC rats, suggesting their potential as biomarkers of bone health in women taking CHC.

**Conclusion:**

Both OCs have adverse effects on various skeletal parameters, with CHC having a greater negative impact on bone strength.

## 1 Introduction

Peak bone mass achievement is critical to bone health and is influenced by various factors, including genetics, physical activity, and nutrition. Genetics plays a significant role, accounting for about 50% of the variation in peak bone mass (1). Females achieve peak bone mass at the femoral neck and hip around 16-19 years, while peak bone mineral density (BMD) at the lumbar spine is achieved around 40-44 years of age (2). Oral contraceptives (OCs), both hormonal and non-hormonal, are widely used by adolescent girls and young women who are still in the process of gaining peak bone mass. Understanding the effects of these contraceptives on bone health is crucial for assessing the potential risk of osteoporosis and fractures in the future.

The impact of OCs on bone health in humans remains uncertain, with conflicting data on whether their use improves (3–7) or impairs (8–15) BMD. Reported studies have determined BMD scores or bone turnover marker (BTM) levels, such as procollagen 1 intact N-terminal propeptide (P1NP) or type I collagen cross-linked C-telopeptide (CTX-1), to investigate the effect of OCs on bone. However, their impacts on bone quality have not been studied. The measures of bone quality include bone architecture, mineralization, and biomaterial composition, and serve as better predictors of bone strength and fracture risk than BMD or BTM alone. Thus, it is crucial to consider both BMD and bone quality when evaluating the impact of OCs on bone health.

In humans, BMD and BTMs are the only feasible parameters for assessing bone health. Investigating bone quality parameters is challenging, as it requires an invasive procedure, such as bone biopsy, which is not feasible for routine clinical practice. Therefore, animal studies are the only means to gain insight into the effects of contraceptives on bone quality and strength, which can be extrapolated to humans.

Two types of OCs are currently available: hormonal and non-hormonal. Hormonal contraceptives include combined hormonal contraceptives (CHC), which contain low doses of estrogen (usually ethinyl estradiol) and progestin, as well as progestin-only contraceptives (16). Centchroman (INN: Ormeloxifene), a non-steroidal compound that acts like a selective estrogen receptor modulator (SERM) and clinically approved in India as a weekly pill, is the sole non-hormonal choice (17). CHCs are more widely used than non-hormonal contraceptives (NHC) by adolescent girls and young women still gaining peak bone mass. Hence, it is essential to understand their potential impact on bone health. The CDC reports that 12% of women aged 15-49 in the US use OCs as their primary method of birth control, and the use is higher among women aged 20-29 years (18). According to the Guttmacher Institute, 62% of sexually active adolescent females aged 15-19 use OCs, and 25% of female college students use OCs as their primary NHC of contraception (19). To understand the long-term impact of OC on bone health, it is crucial to assess adolescent women and follow them until they achieve peak bone mass, which typically occurs in women in their third decade of life.

For this reason, we recruited pubertal female SD rats (6-8 weeks old), which corresponds to the age of pubertal women (~15 years old), and assessed the effect of CHC (levonorgestrel + ethinyl estradiol) and NHC (ormeloxifene), on the bone at a prescribed human dose converted to a rat dose. The rats were treated with OCs for 3- and 7 months, corresponding to approximately 9- and 14-human years, respectively. Once the rats completed the 7-month dosing period, they were 9 months old, which is when rats achieve skeletal maturity and correspond tentatively to the time it takes for humans to reach peak bone mass. After the end of all treatments, we assessed BMD, static and dynamic histomorphometry parameters, bone material composition and bone strength. Through metabolomics, we also studied the changes in metabolites that occurred in the bones and serum of the rats in response to long-term OC treatment. Through this research, our goal is to better understand the potential impact of OCs on bone, which could inform future clinical studies.

## 2 Materials and methods

### 2.1 Animal experiments

Female *Sprague dawley* (SD) rats were procured from the CSIR-CDRI National Laboratory Animal Centre with animal ethics Committee approval no: IAEC/2021/66/Renew-0/Sl no.14 dated 29.06.2021. The animals were kept in cages at 22-25°C with 12h light/dark cycle. During the study, the rats were given ad libitum conventional rodent chow diet and filtered water.

Sixty pubertal female SD rats (age 6-8 weeks) were randomly divided into three groups: one control and two treatment groups (n=20 animals per group). The control group received water as vehicle whereas, the treatment group received either NHC (ormeloxifene, 0.25mg/kg/day; oral) (17, 20) or CHC (levonorgestrel 15.5µg/kg/day and ethinylestradiol 3.1 µg/kg/day; oral) at the dose equivalent to human dose (21). Animals were killed after 3 months and 7 months of dosing. Calcein (20mg/kg; s.c.) was administered to each rat at an interval of 10 days before sacrifice to assess new bone formation (22). Tissue and serum samples were collected and stored at -80˚C until further analysis.

### 2.2 Body composition analysis

Rats were scanned using the EchoMRI™-500 body composition analyzer (EchoMRI Corporation Pvt. Ltd. Singapore). Machine was calibrated before scanning with a known sample of canola oil and distilled water. The fat mass and lean mass were plotted as a percentage of body weight (23)

### 2.3 µCT scanning and analysis

In-life scanning of bone samples was performed using Sky Scan 12726 μ-computed tomography (μCT) scanner (SkyScan, Ltd., Kartuizersweg, Kontich, Belgium) as described before (22). Analysis and quantification was done using CTAn and Batman software respectively. Trabecular and cortical BMD calibration was done from the volume of interest made for the trabecular and cortical region using hydroxyapatite phantom rods of 4 mm of diameter with known BMD (0.25 gHA/cm3 and 0.75 gHA/cm^3^).

### 2.4 Static bone histomorphometry

Goldner’s trichome (GT) staining was done on 7μm sections for histomorphometry on tibia metaphysis as described before (24). Undecalcified bones were preserved in 70% ethanol and methyl methacrylate (MMA) blocks were prepared to determine unmineralized osteoid volume per bone volume (OV/BV), osteoid surface per bone volume (OS/BV) and osteoid width (O.Width) using BioquantOsteo programme (BIOQUANT Software, California, USA).

### 2.5 Dynamic bone histomorphometry

Surface referent bone formation was determined by dynamic histomorphometry on undecalcifed tibial bone sections. Tb. Rodent-VK blue TRAP F1 task list of BioquantOsteo program was used for analysis. 10 days calcein labelling interval was taken into account for the calculations. Mineralizing surface per bone surface (MS/BS), mineral apposition rate (MAR), and bone formation rate per bone surface (BFR/BS), percentage of mineralizing osteoid (MS/OS) and mineralization lag time (MLT) were measured (25) using below given formula:


MAR=Ir.L.Th/10 days(μm/day);

BFR=MAR×(MS/BS)(μm/day);

MS/OS=100*(MS/BS)/(OS/Bs);

MLT=(O.Th*OS/BS)/MAR*MS/BS).

### 2.6 Measurement of serum sex hormone-binding globulin (SHBG)

Serum SHBG was determined by an ELISA kit (catalogue number: E-EL-R0883 Elabscience, USA respectively) following the manufacturers’ protocol. Briefly, this assay employed the Sandwich-ELISA principle, which involved binding of rat SHBG with immobilized antibodies, forming a sandwich with biotinylated detection antibodies and an Avidin-HRP conjugate together. SHBG content in the serum was determined by spectrophotometric measurement at a wavelength of 450 nm by measuring the color change resulting from the enzyme-substrate reaction. The OD values of the supplied standards were used to draw a standard curve, relating OD to known concentrations of SHBG.

### 2.7 Bone and serum metabolomics

Femur metaphysis (30mg) were crushed in liquid N_2_ and further processed. Internal standard, ribitol (0.05 mg/ml) was added and samples were homogenised. Extraction was done in methanol and water (3:1, v/v) and dried under mild N_2_ stream. Then, 30µl of methoxyamine solution (20 mg/ml in pyridine) was added to each vial and incubated at 50˚C for 30 minutes. Finally, 80 µl of MSTFA comprising 1% trimethylchlorosilane (TMCS) was used to derivatize the samples for 45 minutes at 60˚C. After derivatization, 100µl of supernatant was collected and transferred to fresh GC-vials for analysis.

For serum metabolic analysis, 50 µl serum samples was used and extraction procedure similar to bone samples was applied by adding ribitol as internal standard followed by procedures as stated before (26, 27). As part of the pre-processing, MS-Dial version 4.80 was utilized to pick peaks, align, annotate, and deconvolute the mass spectra acquired by GC-MS/MS. After annotation, an open source software metaboanalyst 5.0 were used for statistical analysis.

### 2.8 Bone mechanical testing

#### 2.8.1 Three-point bending

Femur bones were kept in saline-soaked gauze before the testing and 3-point bending test was performed as described before (28). Briefly, femurs were kept between the two roller supports and load (10 N) was applied up to fracture limits at mid-diaphysis. The tests were performed on the electromagnetic testing system (Electroforce 3200; Bose, Eden Prairie, MN, USA). To ensure non-movements of samples, a digital microscope (Dino-Lite 5MP, Taiwan) was attached. The load-displacement data were captured at every 0.01s time intervals. The calculated parameters were maximum load (Fmax, N), stiffness (N/mm), work to failure (N/mm), and post-yield displacement (PYD, mm). The yield point was determined with a 0.2% offset method (29)

#### 2.8.2 Nanoindentation

The femur was cut from mid-diaphysis with a low-speed diamond blade saw (IsoMet; Buehler, Lake Bluff, IL, USA), the cut samples were kept in epoxy for nearly 2 h for proper cureing and, further samples were processed as previously published protocol (28). The experiment was performed on the T1-950 Tribo Indenter (Hysitron Inc., Minneapolis, MN, USA) with Berkovich pyramidal tip in the moist state. Eight indents with a peak load of 1000 µN were applied to cross-section of the bone. The loading segment, holding segment and unloading segment, all were for 10s. The each stage was for 10 Sec. The resultant load-displacement curve were used to calculate the reduced modulus (Er) and hardness (H) by the method of Oliver and Pharr (29–31).

#### 2.8.3 Cyclic reference point indentation (cRPI)

cRPI was performed on the mid-diaphysis of rat femur bone by using BioDent Hfc instrument (Active life scientific, Santa Barbara, CA) with probe BP2 as per the protocol (32). Before and after the experiments on the samples, the probe was tested on the standard polymethylmethacrylate (PMMA) block. For each sample, five indentations were performed and spacing between each indent was 1 mm to avoid the inference of two consecutive indents. The indentation frequency was 2 Hz (40 N/s) with a maximum load of 10 N for 20 cycles.

#### 2.8.4 Measurement of mineral and collagen properties

The mineral and collagen properties were analyzed using Bruker IFS 66v/S FTIR spectro-photometer in the attenuated total reflectance mode, under the constant pressure, in the range of 4000 to 400 cm^-1^ and calculated the following parameters: carbonate to phosphate ratio (area ratio of the carbonate peak [852-890 cm^-1^] to phosphate peak [916-1180 cm^-1^]), mineral to matrix ratio (area ratio of phosphate peak [916-1180 cm^-1^] to the amide-1 peak [1596-1712 cm^-1^]), collagen maturity ratio (intensity ratio of peak [1660 to 1690 cm^-1^]), mineral crystallinity ratio (intensity ratio of peak [1030 to 1020 cm^-1^]), which is related to crystal size and stoichiometric perfection), and acid phosphate content (intensity ratio of [1127 to 1096 cm^-1^]). The Amide I band peak contents number of sub-peak, which gives information about the collagen matrix and the location of cross-linkage and non-cross linkage. The sub-band of the Amide I peak were fitted with Gaussian curves at 1610, 1630, 1645, 1660, 1675, and 1690 cm^-1^ by using peak analyzing tools OriginPro 8.5 software (33, 34).

### 2.9 Statistical analyses

Data were analyzed using Graph Pad Prism 5 and expressed as the mean ± standard error of mean (SEM). Data having more than 2 groups were analysed by one-way ANOVA with Kruskal-Wallis test in case of heterogeneity of variance or Dunnett’s test in case of homogeneity of variance for *post hoc* analysis.

## 3 Results

### 3.1 Effect of OCs on body weight, body composition, bone length and serum SHBG

Treatment of CHC for 3 months reduced body weight in the rats, primarily due to decreased lean mass, although the fat mass was significantly increased compared to the control group. However, after 7 months, there was no significant difference in body weight between the CHC and the control group, even though the fat mass was still greater and the lean mass was lower in the CHC group. On the other hand, NHC had no significant impact on body weight, fat mass, or lean mass at either time point (**Supplementary Figure 1**).

The 3-month NHC treatment significantly increased femur length compared with control; however, there was no significant difference in femur length between the three groups at the later time point (**Table 1**).

**Table 1:** Femur parameters.

Parameter	Control	CHC	NHC	Control	CHC	NHC
	3 Months			7 Months		
**Femur Length (cm)**	3.25 ± 0.042	3.31 ± 0.030	3.4 ± 0.051 *	3.45 ± 0.042	3.4 ± 0.057	3.58 ± 0.030
µCT measurements at femur diaphysis						
**BMD** **(g HA/cm^3^)**	1.57 ± 0.048	1.45 ± 0.032	1.60 ± 0.067	1.51 ± 0.021	1.44 ± 0.045	1.46 ± 0.052
**B.Ar (mm^2^)**	7.09 ± 0.314	8.81 ± 0.574 *	7.38 ± 0.371	7.87 ± 0.420	9.14 ± 0.750	8.14 ± 0.579
**B.Pm (mm)**	22.37 ± 0.613	25.02 ± 1.021	20.86 ± 0.925	22.60 ± 0.944	26.32 ± 2.424	21.86 ± 1.188
**Medullary Area (mm^2^)**	4.72 ± 0.227	5.12 ± 0.245	3.75 ± 0.407	4.54 ± 0.373	4.82 ± 0.347	3.69 ± 0.580
**Endosteal Perimeter (mm)**	9.01 ± 0.425	9.91 ± 0.446	7.84 ± 0.571	9.04 ± 0.473	11.42 ± 1.881	8.53 ± 0.594
Biomechanical strength of femur assessed by 3-point bending test						
**Energy stored** **(N-mm)**	39.34 ± 5.70	46.80 ± 12.56	37.35 ± 4.21	50.59 ± 4.94	32.53 ± 6.07 *	36.12 ± 6.47 *
**Maximum Load (N)**	95.95 ± 5.08	92.61 ± 4.19	103.04 ± 2.53*	116.57 ± 4.67	101.08 ± 6.82	106.56 ± 5.04
**Maximum displacement(mm)**	0.625 ± 0.059	0.576 ± 0.057	0.569 ± 0.051	0.659 ± 0.04	0.487 ± 0.049	0.53 ± 0.063
**Stiffness (N/mm)**	293.03 ± 16.69	286.66 ± 24.52	295.53 ± 7.52	302.49 ± 20.43	349.40 ± 21.25	313.22 ± 8.70
**Young's modulus (GPa)**	5.07 ± 0.290	5.67 ± 0.436	5.55 ± 0.163	5.25 ± 0.186	6.26 ± 0.485	5.43 ± 0.525
**Yield strength (MPa)**	74.04 ± 5.00	74.31 ± 12.33	75.39 ± 4.84	92.94 ± 3.35	82.14 ± 6.03	93.88 ± 3.05
**Modulus of Toughness (MPa)**	3.76 ± 0.465	4.37 ± 1.35	3.28 ± 0.377	4.40 ± 0.423	2.85 ± 0.536	3.17 ± 0.596
**Ultimate stress(MPa)**	133.35 ± 7.31	124.18 ± 5.61	138.66 ± 3.50	154.59 ± 6.23	135.29 ± 9.24 *	142.57 ± 6.85
**Resiliance (MPa)**	0.504 ± 0.044	0.581 ± 0.203	0.495 ± 0.067	0.762 ± 0.029	0.155 ± 0.063 **	0.728 ± 0.039
**Fracture point stress (MPa)**	127.142 ± 6.88	120.27 ± 7.05	134.26 ± 5.61	147.76 ± 5.13	134.73 ± 9.28	141.97 ± 6.31
**Fracture strain(mm/mm)**	0.040 ± 0.003	0.038 ± 0.003	0.037 ± 0.003	0.043 ± 0.003	0.032 ± 0.003	0.034 ± 0.004

BMD, bone mineral density; B.Ar, bone area; B.Pm, bone perimeter. Data are expressed as mean±SEM (n =6). *p<0.05, and **p<0.01 compared with age-matched controls.

As SHBG has critical roles in maintaining the proper balance and the bioavailability of sex hormones, including testosterone, dihydrotestosterone and estradiol, as well as being implicated in bone metabolism (35, 36), we assessed the effects of OCs on serum SHBG levels. Both OCs decreased SHBG after 3 months but increased it after 7 months (**Supplementary Figure 1**).

### 3.2 Effect of OCs on bone mass and microarchitecture

**Figure 1:**
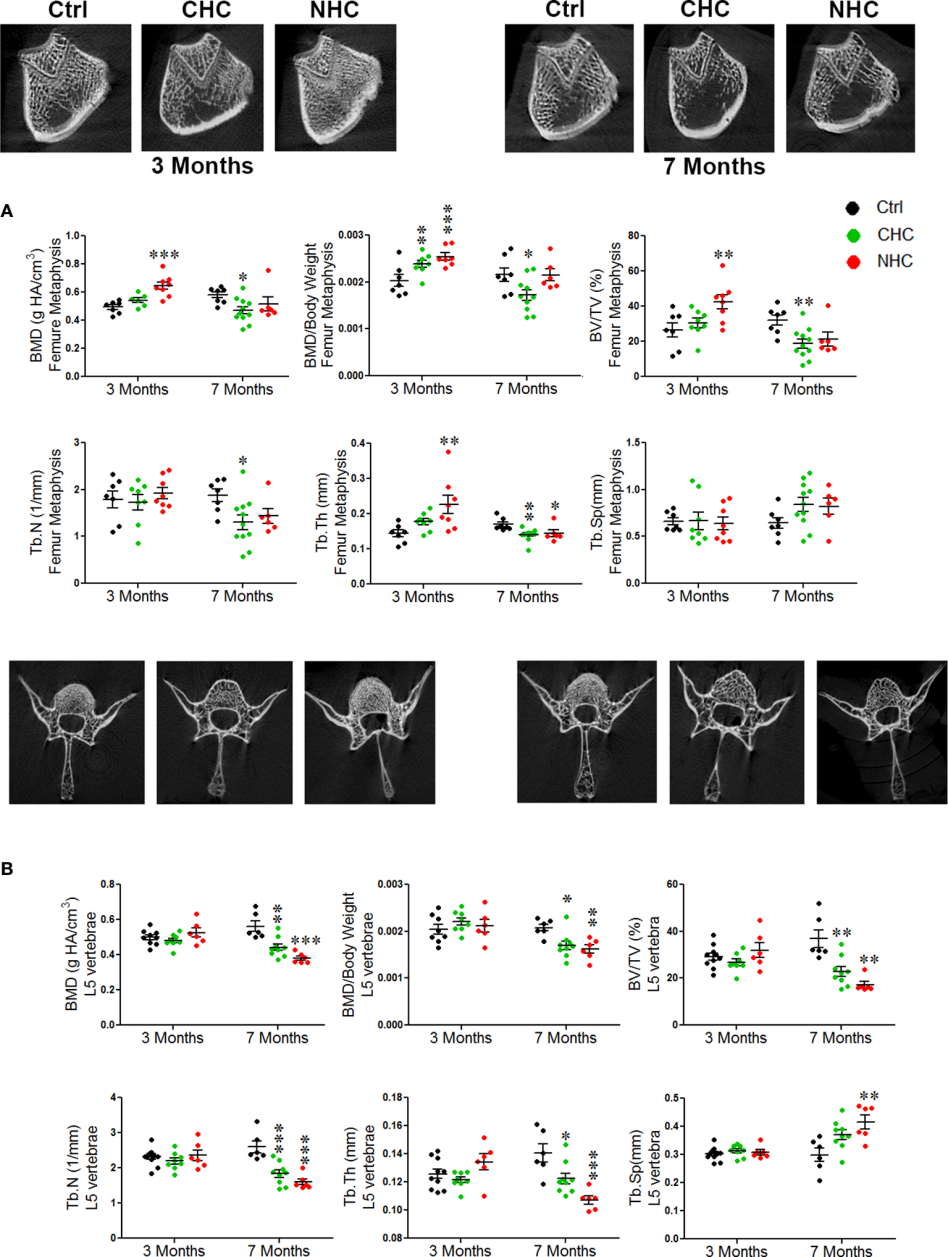
Effect of OCs on bone mass and microarchitecture assessed by µCT; **(A)** femur metaphysis (upper pane - representative image and lower panel - quantification of the parameters) and **(B)** L5 vertebrae (upper panel - representative images and lower panel - quantification of various parameters). Data are expressed as mean ± SEM (n > 6 rats/group). *p<0.05, **p<0.01 and ***p<0.001 compared with the age-matched control (vehicle treated). Ctrl, control; CHC, combined hormonal contraceptive; NHC, non-hormonal contraceptive.

After 3 months of treatment, CHC did not affect BMD and trabecular microarchitecture in the metaphysis of the long bones and L5 (**Figure 1A**). On the other hand, NHC significantly increased BMD, weight-normalised BMD, BV/TV, and Tb.Th in femur metaphysis compared to the control, but did not affect L5 vertebral parameters. Notably, when weight-normalized BMD of femur metaphysis was considered, CHC had higher values than the control (**Figure 1A**). In tibia metaphysis, CHC did not affect trabecular microarchitecture except Tb.Th, which was increased compared with control. On the other hand, NHC treatment after 3 months increased BMD, weight-normalized BMD, BV/TV and Tb.Th compared with control whereas Tb.N and Tb.Sp were comparable between the two groups (**Supplementary Figure 2**).

After 7 months of treatments, the gains in bone mass and trabecular microarchitecture observed after 3 months with NHC at femur metaphysis were lost as all parameters were comparable with age-matched control except Tb.Th, which was lower than control (**Figure 1A**). In tibia, however, all parameters except weight-normalized BMD and Tb.Sp were lower than control (**Supplementary Figure 2**). In the CHC group, all femoral parameters were lower than the control except Tb.Sp (**Figure 1A**). In tibia metaphysis, the extent of impairment by CHC was similar to femur as BMD, BV/TV and Tb.N were reduced and Tb.Sp was increased compared with control (**Supplementary Figure 2**). The most pronounced negative effect was seen in L5 by NHC as all parameters showed deterioration compared with control (**Figure 1B**). In case of CHC, all parameters except Tb.Sp were lower than the control (**Figure 1B**).

When cortical bone was studied using femur diaphysis, BMD and geometric parameters were comparable between the OCs and the corresponding age-matched control except bone area after 3 months of CHC treatment that was higher than the age-matched control (**Table 1**).

### 3.3 Effect of OCs on bone formation and mineralization

**Figure 2:**
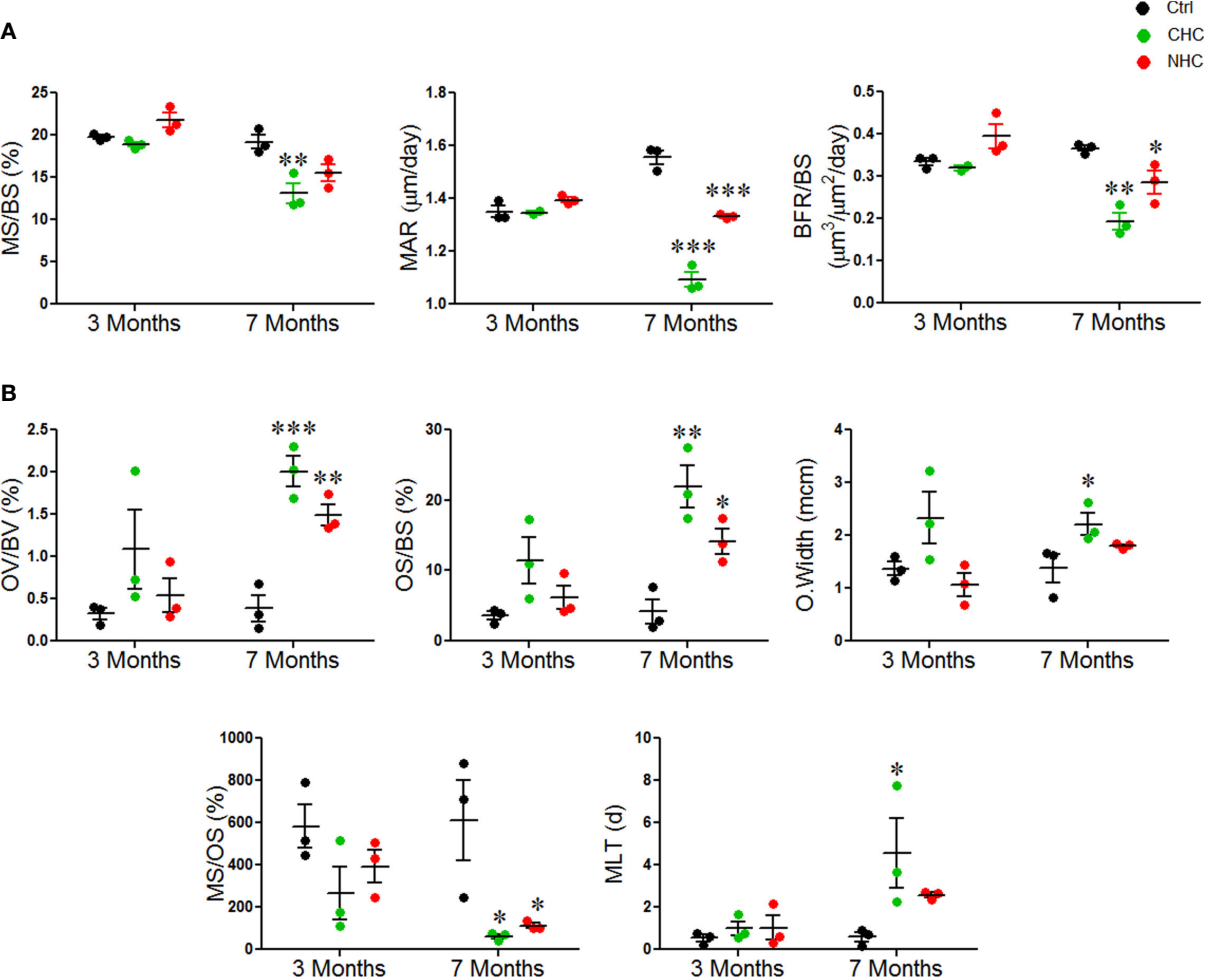
Effect of OCs on bone formation and mineralization; **(A)** dynamic histomorphometry parameters and **(B)** parameters derived from GT staining. Data are expressed as mean ± SEM (n = 3 rats/group). *p<0.05, **p<0.01 and ***p<0.001 compared with the age-matched control. Ctrl, control; CHC, combined hormonal contraceptive; NHC, non-hormonal contraceptive.

We used time-spaced fluorochrome labeling to measure surface-referent bone formation. After three months of treatments, MS/BS, MAR, and BFR/BS were comparable between CHC, NHC, and control. After 7 months of treatments, CHC significantly decreased MS/BS compared to the control group, indicating a decrease in the bone-forming surface. Additionally, both drugs significantly reduced MAR and BFR/BS, indicating a decrease in the rate of mineral accretion and the average amount of bone formed by a team of osteoblasts per activation event at the remodeling site, respectively (**Figure 2A**).

GT staining was next used to evaluate the mineralization and collagen content of the bone tissue. After 3 months, osteoid volume per bone volume (OV/BV), osteoid surface per bone surface (OS/BS), osteoid width (O.width), mineralizing surface per osteoid surface (MS/OS), and mineralization lag time (MLT), were comparable between the two drug groups and the control group. After 7 months, both drugs significantly increased OV/BV and OS/BS compared to the control group, and CHC significantly increased O.width. Both drugs also significantly decreased the percentage of MS/OS after 7 months, indicating a decrease in the amount of mineralized osteoid surface. Additionally, CHC dramatically increased MLT at 7 months, indicating a delay in the mineralization process (**Figure 2B**). Overall, these results revealed that the OCs diminished bone formation and mineralization and that the negative impact of CHC was more than NHC.

### 3.4 Effect of OCs on bone strength

The 3-point bending test was used to assess the strength and mechanical properties of femur diaphysis. After 3 months of treatment, there was no significant difference in the mechanical properties between CHC and control groups. However, the maximum failure load was significantly higher in the NHC group compared to the control group. After 7 months, both drugs reduced the energy storage capacity, but CHC also caused a decrease in ultimate stress and resilience compared to the control (**Table 1**).

**Figure 3:**
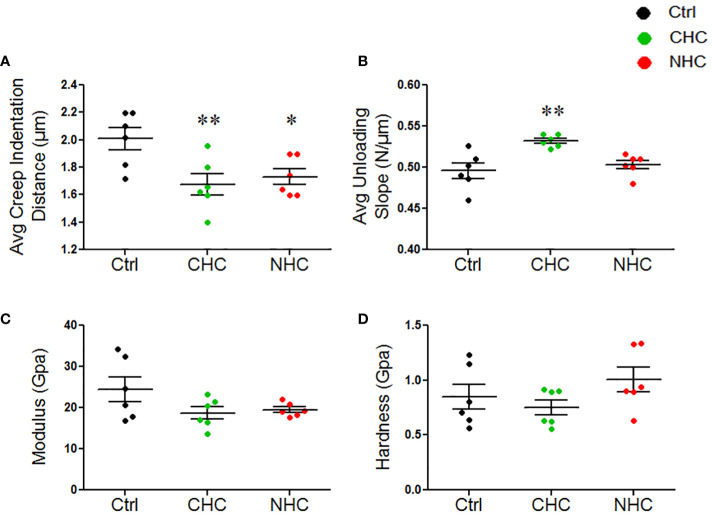
Effect of OCs on mechanical properties of bones at the micro- and nanoscale; **(A)** Average creep indentation distance. **(B)** Average unloading slop. **(C)** Modulus, **(D)** Hardness. Data are expressed as mean ± SEM (n=6 rats/group). *p<0.05, and **p<0.01 compared with the age-matched control. Ctrl, control; CHC, combined hormonal contraceptive; NHC, non-hormonal contraceptive.

cRPI is a specialized multi-axial microindentation technique (37) used to study the mechanical properties of bone tissue to cyclic loading that occurs during walking or running. cRPI involves applying a series of small loads (on the order of milli-Newtons) to the bone tissue and measuring its deformation in response to each load. Analysis of the bone tissue’s deformation behavior over time enables insight into the tissue’s mechanical properties, including its ability to withstand repetitive loading. After 3 months, there were no significant differences in any of the cRPI measures, including total indentation distance, creep indentation distance, average energy dissipated, average unloading slope, and average loading slope between either OC and the control group (data not shown). After 7 months, both OCs reduced creep indentation distance compared with the control (**Figure 3A**). Besides, CHC had a higher average unloading slope than the control (**Figure 3B**). There were no differences in total indentation distance, average energy dissipated, and average loading slope between either OC and the control group after 7 months (data not shown).

As opposed to cyclical load in cRPI, nanoindentation uses a continuous uniaxial force to indent the bone, typically at minimal loads (on the order of micro-Newtons), to measure the force and displacement at the indentation depth at a specific point in time (38). Analyses of the force-displacement curve generated during the indentation process allow the determination of the elastic modulus and hardness of the bone lamellae. This information can provide insights into the material properties and mechanical behaviour of bone at the microstructural level. We observed no significant differences in the modulus and hardness at both treatment times by either OC (**Figures 3C, D**).

### 3.5 Effect of OCs on bone material composition

**Figure 4:**
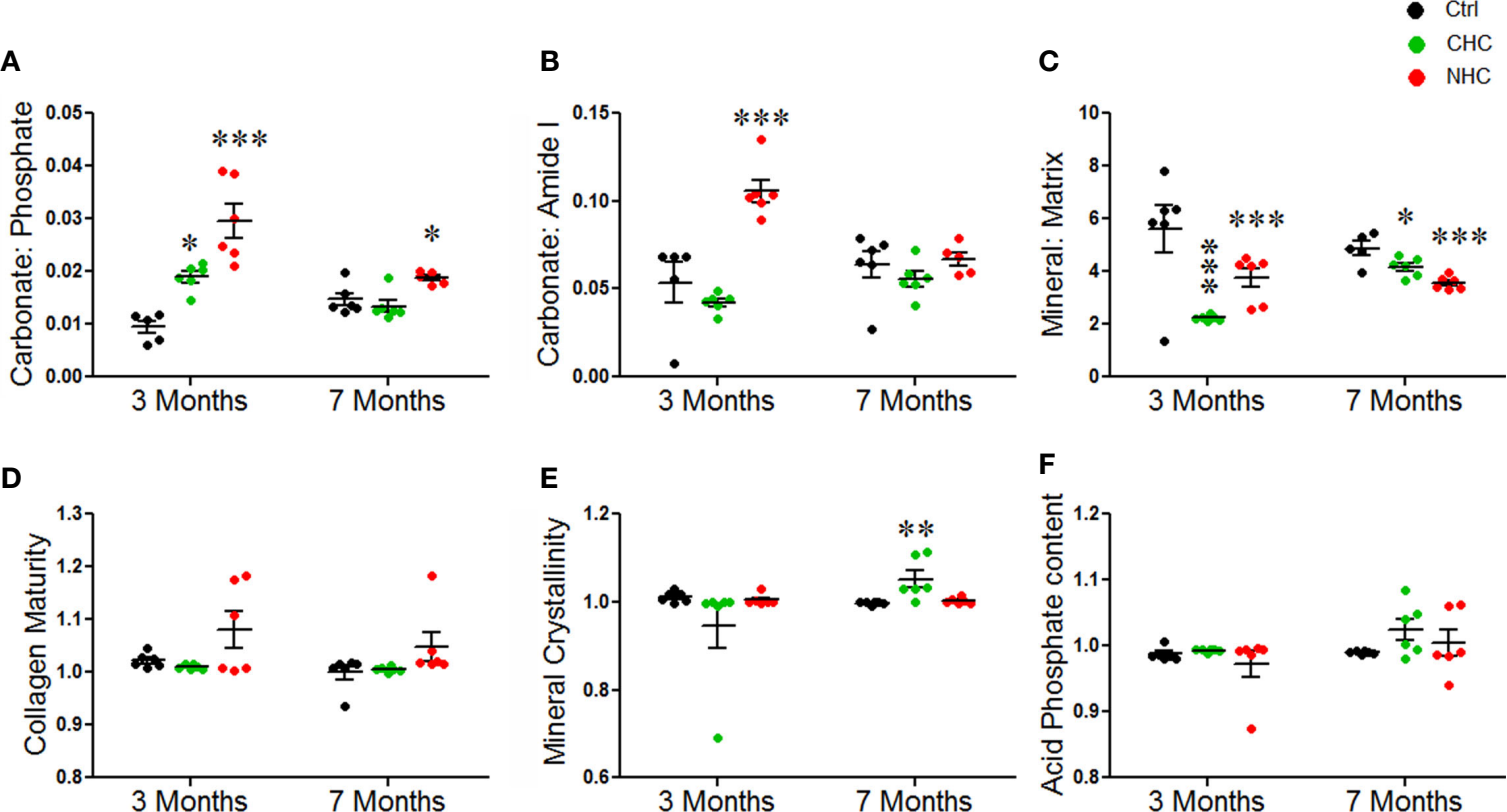
Effect of OCs on bone material composition assessed by FTIR. **(A)** Carbonate to phosphate ratio. **(B)** Carbonate to amide I ratio. **(C)** Mineral to matrix ratio. **(D)** Collagen maturity. **(E)** Mineral crystallinity. **(F)** Acid phosphate content. Data are expressed as mean ± SEM (n = 6 rats/group). *p<0.05, **p<0.01 and ***p<0.001 compared with vehicle of corresponding time point. Ctrl, control; CHC, combined hormonal contraceptive; NHC, non-hormonal contraceptive.

We next investigated the effects of OCs on the material composition of bones using FTIR spectroscopy on bone powder samples. The carbonate/phosphate ratio and carbonate/amide I ratio were used to measure how much carbonate had substituted phosphate (inorganic component) or amide (organic component) of bone matrix, respectively. After 3 months of treatment, the carbonate/phosphate ratio was increased in both OC groups. This effect persisted after 7 months only in the NHC group, suggesting that the changes induced by NHC might be more long-lasting than those induced by CHC (**Figure 4A**). The carbonate/amide I ratio was increased by NHC after 3 months, indicating increased hydroxide substitution in the apatite crystal (**Figure 4B**). However, after 7 months, this ratio was comparable between all groups.

The mineral/matrix ratio, which represents the amount of mineral (phosphate) content relative to the collagen matrix (amide I), was decreased by both OCs at both time points (**Figure 4C**). This suggests that the bone was losing mineral content, which could affect its strength and resilience. However, the collagen maturity, mineral crystallinity, and acid phosphate content remained unchanged after 3 months of treatment with either OC. After 7 months, mineral crystallinity was significantly increased in the CHC group, while collagen maturity and acid phosphate content were comparable between all the groups (**Figures 4D–F**).

### 3.6 Effects of OCs on bone and serum metabolome

Studying both bone and serum metabolomes in response to OCs is vital because it provides a comprehensive understanding of their effects on bone health. By examining both bone and serum metabolome, it is possible to identify potential biomarkers of drug efficacy and safety and gain insight into the underlying mechanisms of action of the OCs.

Univariate and multivariate statistical analysis showed substantial metabolic changes in bone and serum samples of rats treated with OCs compared to the control. Bone and serum samples had 176 and 154 annotated metabolites, respectively. In control, between 3- and 7 months, there were changes in the 41 and 98 metabolite levels in bone and serum, indicating these may be age-related and excluded from further statistical analysis.

**Figure 5:**
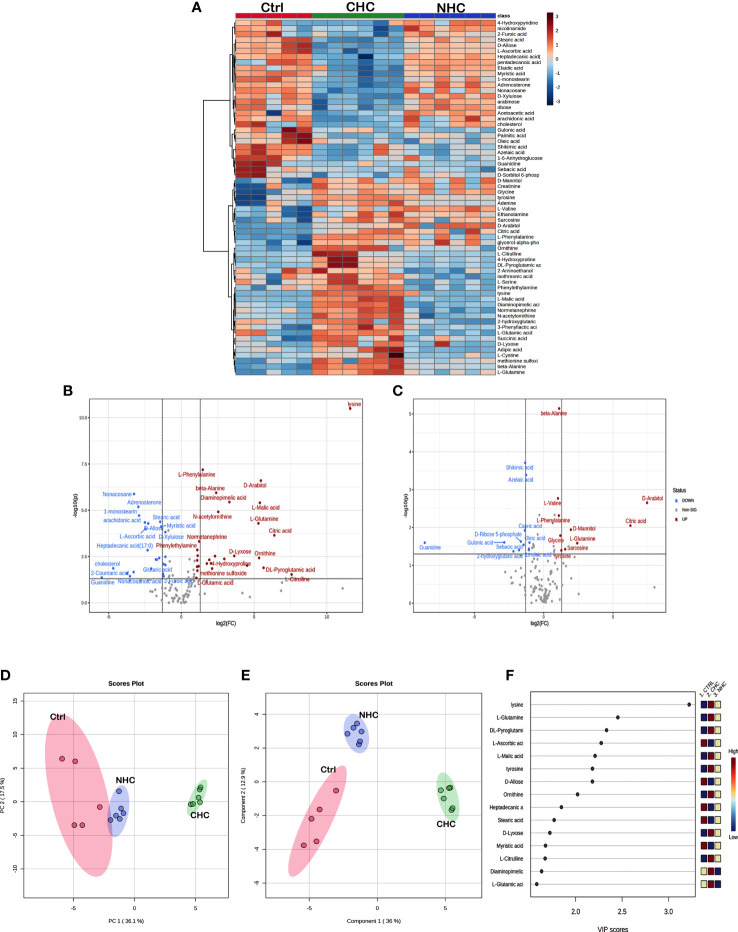
Effect OCs on bone metabolome. **(A)** Heat Map of 63 significantly altered metabolites (p<0.05) with CHC and NHC treatments after 7 months. Volcano plot depicting aberrantly regulated metabolites with p< 0.05 and fold change of 2.0 after seven months of CHC **(B)** or NHC **(C)** treatment compared with control. **(D)** Overview of untargeted metabolomics results. PCA score plot for the metabolites detected in positive ion mode; circles represent the 95% confidence interval. **(E)** A partial least square-discriminant analysis (PLS-DA) score plot for separation of different groups. **(F)** Variable importance in projection (VIP) score of PLS-DA. Colour boxes on the right indicate the relative concentration of corresponding metabolites. (n> 5 samples/group). Ctrl, control; CHC, combined hormonal contraceptive; NHC, non-hormonal contraceptive.

**Figure 6:**
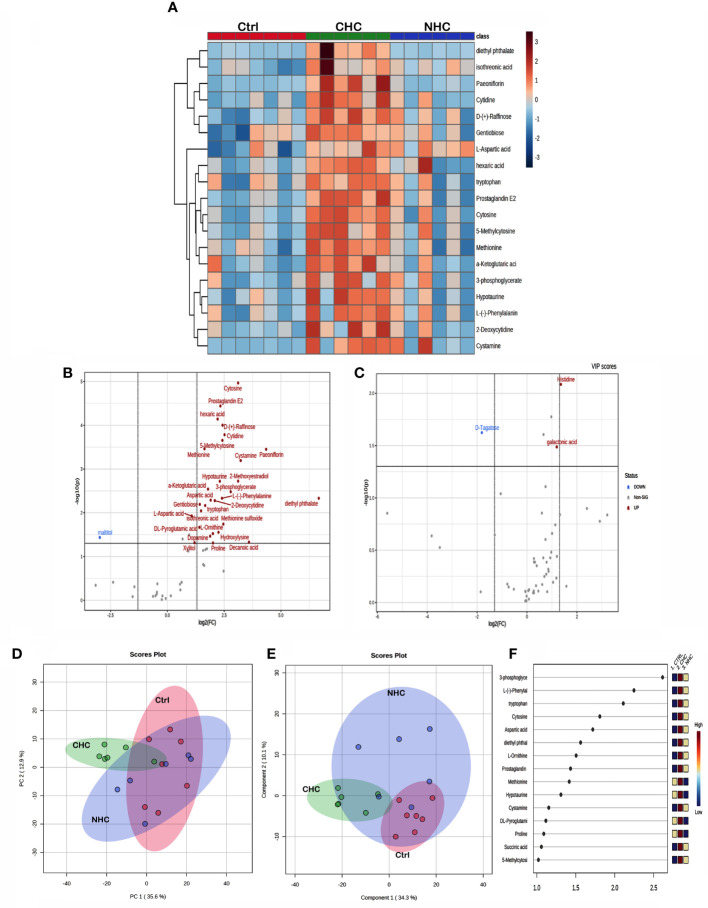
Effect OCs on serum metabolome. **(A)** Heat map showing the total number of metabolites in serum altered (p<0.05) after 7 months of CHC or NHC treatment. Volcano plot depicting aberrantly regulated metabolites with p< 0.05 and fold change of 2.0 after seven months of CHC **(B)** or NHC **(C)** treatment compared with control. **(D)** PCA score plot; circles represent the 95% confidence interval. **(E)** PLS-DA score plot and **(F)** VIP score of PLS-DA where colour boxes on the right indicate the relative concentration of corresponding metabolites. (n> 5 samples/group). Ctrl, control; CHC, combined hormonal contraceptive; NHC, non-hormonal contraceptive.

The univariate ANOVA *post hoc*-test revealed that 63 and 19 metabolites were changed in the bone (**Figure 5A**) and serum (**Figure 6A**) of OC-treated groups compared with the control group (p< 0.05). Next, we utilized a volcano plot to identify metabolites that differed significantly between control vs. CHC and control vs. NHC (p< 0.05, fold change >2.0). 28 metabolites were increased in the CHC-treated bones, and 24 decreased compared with control (**Figure 5B**). In the serum, 29 metabolites were increased, and one metabolite was decreased compared with the control (**Figure 6B**). Relatively, the changes in the number of metabolites in the NHC group compared with the control; 10 were increased, and 11 were decreased in the bone (**Figure 5C**); 2 were increased, one was decreased in the serum (**Figure 6C**).

Unsupervised PCA and supervised PLS-DA were used for multivariate exploratory analysis in bone and serum samples. PC1 and PC2 accounted for 36.1% and 17.5% in bone tissue and 35.6% and 12.9% of the variability in serum, respectively (**Figures 5D**, **6D**). PLS-DA improved group separation with a CI of 95% (**Figures 5E**, **6E**). VIP analysis identified the top 15 drug-induced altered metabolites have potential role in PLS-DA group separation in both bone and serum samples (**Figures 5F**, **6F**). Cross-validation tested model robustness and data repeatability. The model was resilient and reproducible since the cross-validation coefficient R2 (0.968 & 0.999) and Q2 (0.481 & 0.955) of serum and bone respectively showed the quality of fit (raw data **Supplementary Files 1, 2**).

### 3.7 Metabolic pathway analysis and biomarker identification

**Figure 7:**
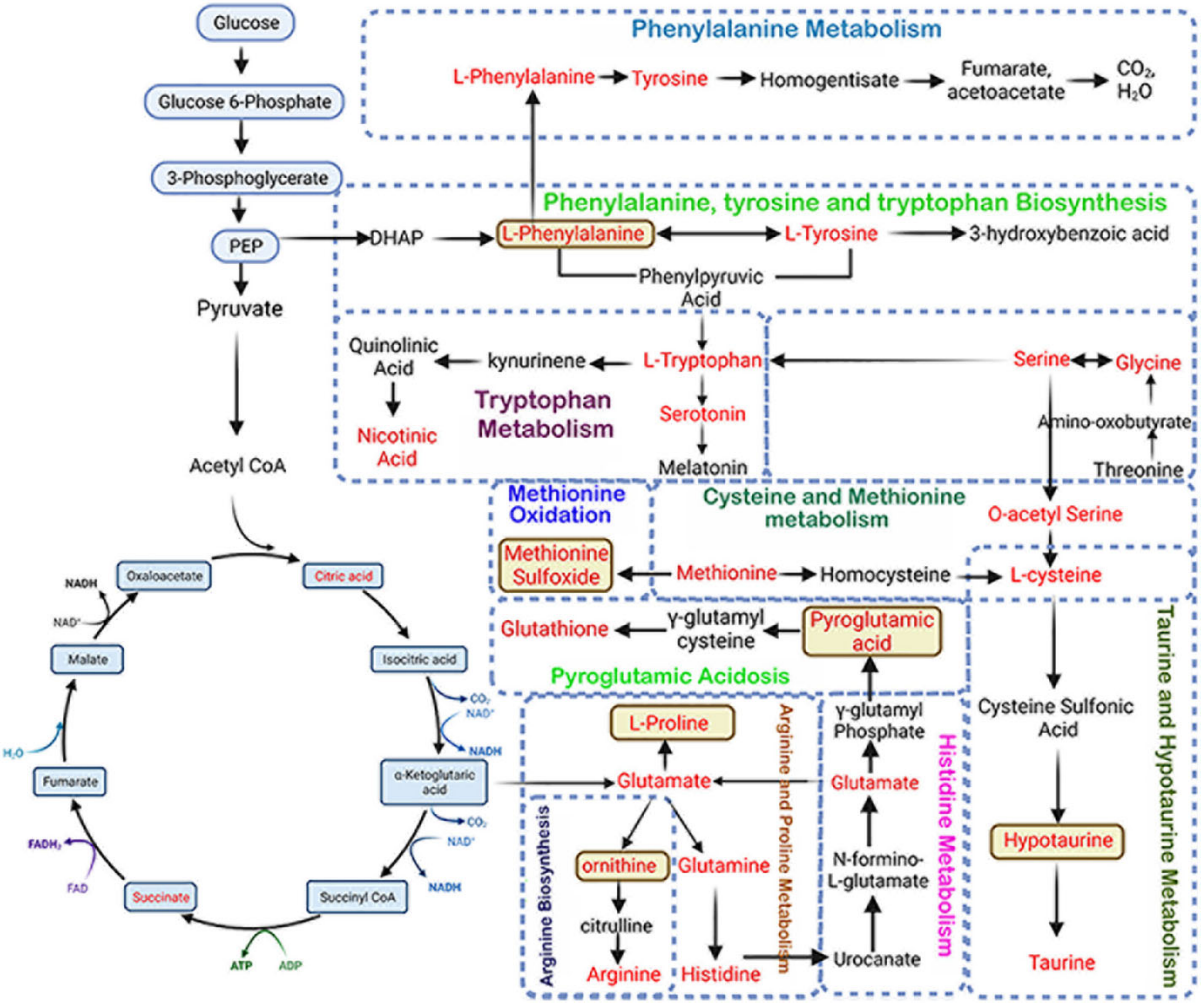
Schematic representation of altered metabolic pathways in bone and serum by CHC after 7 months. L-phenylalanine, methionine sulfoxide, pyroglutamic acid, L-proline, ornithine, and hypotaurine are potential biomarkers as these metabolites are increased in both serum and bone.

We conducted a KEGG metabolic pathway analysis to identify the impact of CHC and NHC on bone and serum metabolites. After 7 months of CHC treatment, 24 metabolic pathways in bone and 9 metabolic pathways in serum were significantly altered (p< 0.05, impact >0.05). Seven metabolic pathways were upregulated in both serum and bone in response to CHC. These pathways include phenylalanine, tyrosine, tryptophan, and arginine biosynthesis; and taurine, hypotaurine, phenylalanine, arginine, proline, alanine, aspartate, glutamate, cysteine, and methionine metabolism (**Figure 7**, **Table 2**).

**Table 2:** Altered metabolic pathways after 7 months of CHC treatment in bone and serum.

Pathway Name	CHC (Femur)	CHC (Serum)
**Phenylalanine, tyrosine and tryptophan biosynthesis**	Up regulated (Phenyalanine & Tyrosine ↑)	Up regulated (Phenylalanine ↑)
**Taurine and hypotaurine metabolism**	Up regulated (Taurine, hypotaurine & cysteine ↑)	Up regulated (Taurine & Hypotaurine ↑)
**Synthesis and degradation of ketone bodies**	Up regulated (3-hydroxybutanoate ↑)	-
**Phenylalanine metabolism**	Up regulated (Phenylalanine, tyrosine and phenylethlamine ↑)	Up regulated (L-phenylalanine ↑)
**Glycine, serine and threonine metabolism**	Up regulated (glycine, serine, cysteine and sarcosine ↑)	-
**D-Glutamine and D-glutamate metabolism**	Up regulated (glutamine and glutamate ↑)	–
**Beta-Alanine metabolism**	Up regulated (5,6 dihyrouracil, beta-alanine ↑)	-
**Arginine biosynthesis**	Up regulated (glutamate, glutamine, citrulline, ornithine, acetyl ornithine ↑)	Up regulated (2-oxoglutarate, aspartate, glutamate and ornithine ↑)
**Arginine and proline metabolism**	Up regulated (proline, hydroxyproline, glutamate, ornithine ↑)	Up regulated (ornithine, proline and glutamate ↑)
**Arachidonic acid metabolism**	Down regulated (Arachidonic acid **↓)**	–
**Alanine, aspartate and glutamate metabolism**	Up regulated (Citrate, succinate, glutamate, glutamine, alanine and asparagine↑)	Up regulated (Aspartate, citrate, glutamate, 2-oxoglutarate and succinate ↑)
**Tyrosine metabolism**	Up regulated (Tyrosine, dopamine, norepinephrine ↑**)**	–
**Pentose and glucuronate interconversions**	Down regulated (Xylose, xylulose and Gulonate **↓)**	-
**Citrate cycle (TCA cycle)**	Up regulated (malate, cis-aconitate, citrate and succinate ↑)	–
**Glyoxylate and dicarboxylate metabolism**	Up regulated (Citrate, cis-aconitate, malate, glutamate, glutamine, Glycine, Serine ↑)	-
**Pentose phosphate pathway**	Down regulated (Ribose and Ribose five phosphate **↓)**	–
**Pyrimidine metabolism**	Up regulated (Glutamine, 5,6 dihuydrocuracil, beta alanine, thymine ↑)	-
**Glutathione metabolism**	Up regulated (Cysteine, 5-oxoproline, glutamate, DL-Pyroglutamic acid, glycine and ornithine ↑)	–
**Cysteine and methionine metabolism**	Up regulated (serine, cysteine and cysteine ↑)	Up regulated (Methionine ↑)
**Butanoate metabolism**	Up regulated (glutamate, succinate, 3-hydroxy butanoates ↑**)**	–
**Purine metabolism**	Up regulated (adenine, guanosine, guanine, urate & xanthine ↑**)**	-
**Primary bile acid biosynthesis**	Down regulated (cholesterol **↓)**	–
**Amino sugar and nucleotide sugar metabolism**	Down regulated (N-acetyl D- mannosamine **↓)**	-
**Aminoacyl-tRNA biosynthesis**	–	–
**Histidine metabolism**	-	Up regulated (Histidine, glutamate and aspartate ↑)
**Tryptophan metabolism**	–	Up regulated (tryptophan ↑)

We also identified 52 metabolites in bone and 30 in serum that showed more than 2-fold accumulation after 7 months of CHC treatment compared to the control. Six metabolites were found to be common in both serum and bone, including DL-pyroglutamic acid, ornithine, methionine sulfoxide, L-phenylalanine, hypotaurine, and L-proline (**Figure 7**). These metabolites were significantly higher in both bone and serum after 7 months of CHC treatment, suggesting that they may serve as biomarkers vis-à-vis bone health in response CHC treatment.

After 7 months of NHC treatment, 17 metabolic pathways in bone were significantly altered, but none in serum (p< 0.05, impact >0.05). These pathways that were altered in bone include phenylalanine, tyrosine, tryptophan, arginine, and aminoacyl-tRNA biosynthesis; and linoleic acid, phenylalanine, glycine, serine, threonine, alanine, β-alanine, aspartate, glutamate, tyrosine, purine, pyrimidine, glutathione, nicotinate, nicotinamide, vitamin B6 glyoxylate and dicarboxylate metabolism. Additionally, pathways related to pentose and glucuronate interconversions, and TCA cycle were also affected (**Table 3**).

**Table 3:** Altered metabolic pathways after 7 months of NHC treatment in bone and serum.

Pathway Name	NHC (Femur)	NHC (Serum)
**Phenylalanine, tyrosine and tryptophan biosynthesis**	Up regulated (Phenylalanine and Tyrosine ↑)	-
**Linoleic acid metabolism**	Down regulated (linoleic acid **↓)**	–
**Phenylalanine metabolism**	Up regulated (phenylalanine and tyrosine ↑)	-
**Glycine, serine and threonine metabolism**	Up regulated (Glycine, sarcosine, guanidinoacetate, cysteine ↑**)**	–
**Beta-Alanine metabolism**	Up regulated (5,6 dihydrouracil, uracil and beta alanine ↑)	-
**Arginine biosynthesis**	Down regulated (citrulline, ornithine and acetylornithine Urea **↓)**	–
**Alanine, aspartate and glutamate metabolism**	Up regulated (citrate, glutamine and succinate ↑)	-
**Tyrosine metabolism**	Up regulated (Tyrosine ↑, Normetanephrine **↓)**	–
**Pentose and glucuronate interconversions**	Down regulated (Gulonate **↓)**	-
**Aminoacyl-tRNA biosynthesis**	–	–
**Citrate cycle (TCA cycle)**	Up regulated (Citrate and succinate ↑)	-
**Glyoxylate and dicarboxylate metabolism**	Up regulated (Citrate, succinate, Glutamine, glycine ↑)	–
**Nicotinate and nicotinamide metabolism**	Up regulated (nicotinate and nicotinamide ↑)	-
**Pyrimidine metabolism**	Up regulated (Glutamine, thymine, beta alanine, 5,6 dihydrouracil and uracil ↑)	–
**Glutathione metabolism**	Up regulated (Glycine, ornithine, cysteine ↑)	-
**Purine metabolism**	Up regulated (adenine and glutamine ↑; ribose 5 phosphate **↓)**	–
**Vitamin B6 metabolism**	Down regulated (Lower pyridoxamine **↓)**	-

**Figure 8:**
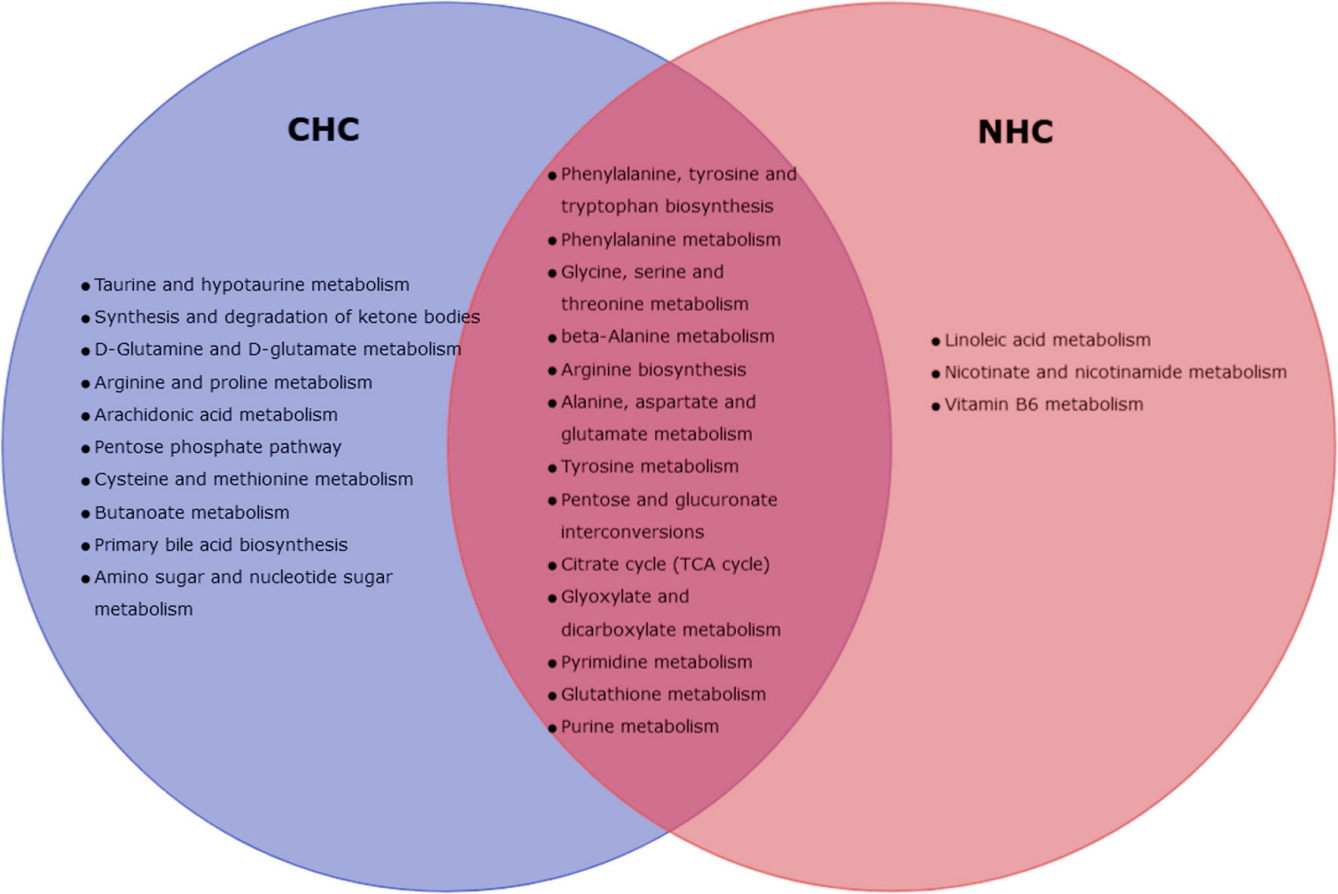
Venn-diagram representing altered metabolic pathways in bone with CHC and NHC after 7 month.

We further observed metabolic pathways altered by both OCs in bone tissue after 7 months of treatment that were similar. These pathways include those involved in the biosynthesis and catabolism of amino acids such as phenylalanine, tyrosine, tryptophan, glycine, serine, and threonine, as well as pathways related to the biosynthesis of alanine, β-alanine, arginine, aspartate, and glutamate. Furthermore, both OCs altered the metabolic pathways related to tyrosine breakdown, pentose and glucuronate conversion, TCA cycle, glyoxylate and dicarboxylate metabolism, purine and pyrimidine metabolism, and glutathione metabolism (**Figure 8**).

## 4 Discussion

Studies investigating the effect of OCs on bone health have yielded mixed results. Some suggest that OC use may improve BMD, while others suggest that it may have a negative effect on bone health, especially in adolescent women. According to a review of published literature, OC use may have little effect or may even be beneficial for bone health in women who have attained skeletal maturity, but it may have a negative effect on adolescent women as they are still growing (39). We found that CHC had no effect on bone mass after 3 months, whereas NHC enhanced BMD in long bones. However, the gain in bone mass was lost with prolonged treatment of NHC, and the CHC treatment resulted in a significant decrease in long bone BMD, likely due to significant increases in SHBG levels by both OCs. The implication of NHC increasing bone mass in long bones after 3 months but losing its effect after 7 months of treatment suggests that there may be a potential window of opportunity for treatment with this OC to maximize its effect on bone mass. Lumbar BMD remained comparable to the control group at 3 months for both OCs but decreased at a later time point, which is consistent with the loss of spinal BMD observed in adolescent women taking OCs (2). Moreover, both OCs caused deterioration of trabecular microarchitecture after 7 months of treatment, indicating the importance of including trabecular bone score (TBS) as a measurement in future studies to investigate whether the decreased BMD observed in young women using OC is also accompanied by reduced TBS, which could potentially inform clinical recommendations for young women considering the use of OC.

The findings from the static- and dynamic histomorphometry measurements suggest that both OCs reduced the activity of osteoblasts after 7 months of treatment. Specifically, CHC reduced the bone formation as indicated by the decreased MS, MAR, and BFR. Additionally, CHC caused delayed osteoid mineralization, which is indicated by the increased osteoid surface, volume, width, and MLT. NHC also showed impaired bone formation, as indicated by the reduced MAR and BFR, and impaired mineralization indicated by increased osteoid volume and surface after 7 months. These results suggest that both OCs have a negative impact on bone formation and mineralization that led to reduced bone mass. Based on these data, it is recommended to conduct human studies to investigate the effects of OCs on bone formation and mineralization. The studies could evaluate the changes in static- and dynamic histomorphometry measurements in women taking the OCs over a period of time, as well as other parameters such as BMD, bone turnover markers, and fracture risk.

After 7 months of treatment with CHC, the 3-point bending experiment revealed decreases in energy storage, resilience, and ultimate stress, implying a reduced capacity of the bone to absorb impacts and increased susceptibility to micro-damage in response to repetitive load. In contrast, NHC only decreased energy storage without any changes in other strength parameters, making it less susceptible to micro-damage and fracture compared to CHC. In contrast to the tissue level changes observed with OCs, particularly with CHC, nano-indentation experiments revealed no change in elastic modulus and toughness which suggested that the OCs did not affect bone’s intrinsic material properties but likely had an effect on the macroscopic properties including overall structure or composition of the bone.

It is interesting to note that cRPI and 3-point bending data showed opposite effects of OCs on bone strength and resistance to microdamage. CHC decreased creep indentation distance and increased average unloading slope which are indicators of increased bone mass and stiffness, respectively. NHC decreased creep indentation distance indicating a lower risk of microdamage. Creep indentation distance is a measure of how much the bone deforms under a constant load over time (40), and a decrease in creep indentation distance indicates that the bone is better able to resist deformation. Similarly, an increase in average unloading slope indicates that the bone is able to better recover its shape after being deformed, which also suggests a lower risk of microdamage (41). Although, CHC decreased the bone’s ability to absorb impacts and increased susceptibility to microdamage, but it retained the ability to repair microdamage and withstand cyclic loading, indicating that a decrease in mineralization likely impaired the bone’s mechanical properties under static loading, but not its ability to withstand cyclic loading over time.

Both OCs increased the carbonate/phosphate ratio after 3 months, suggesting a decrease in the hardness of the mineral phase, as well as an increase in the solubility of the bone mineral. Moreover, an increase in mineral crystallinity as observed in case of CHC, suggests the formation of homogenized mineral crystals, potentially making the bone brittle. Additionally, NHC substantially increased the carbonate/amide I ratio, which reflects the degree of substitution of the mineral phase by non-collagenous proteins. The increased carbonate/phosphate and carbonate/amide I ratios suggest a disordered hydroxyapatite crystal structure due to carbonate incorporation, making the mineral more prone to dissolution or resorption. However, these changes were largely reversed after 7 months, indicating that the initial changes (after 3 months) triggered the demineralization process that culminated in hypomineralized osteoid by both drugs after 7 months. Both OCs decreased the mineral/matrix ratio at both time points and was complemented by a reduction in the amount of mineralized bone, as seen in the GT staining data after 7 months. The OCs had no effect on collagen maturity or acid phosphate levels, implying that these parameters did not mediate changes in mineralization and bone strength.

CHC treatment altered several bone and serum metabolites after 7 months compared to the control. Six metabolites are common in bone and serum, including DL-pyroglutamic acid, ornithine, methionine sulfoxide, L-phenylalanine, hypotaurine, and L-proline. PTH by stimulating ornithine decarboxylase in osteoblasts produces osteogenic polyamines such as putrescine, spermidine, and spermine (42). Ornithine levels are higher in the bones of OVX rats, and we observed the same in response to both OCs, implying that ornithine had an anti-osteogenic effect. Hyperphenylalaninemia, known to inhibit osteogenic differentiation and mineralization of MSC, was increased in bones treated with both OCs, suggesting a potential link to the observed decrease in mineralization by the OCs. Both OCs increased the bone methionine sulfoxide levels, indicating that they accelerated bone aging due to oxidative stress and aging. Accumulation of hypotaurine and proline indicated poor bone mass, consistent with previous studies on osteopenic humans and OVX rats. Higher levels of glutamine and glutamate metabolism have been linked to reduced BMD in women. The increased bone glutamate levels by both OCs imply a negative effect on bone health, as glutamine metabolism is critical in controlling MSC growth and differentiation to osteoblasts. Six metabolites that were increased in serum besides bones of CHC-treated rats but not in serum of NHC-treated rats suggested their potential use as serum biomarkers for bone health in women taking CHC. The more significant overall changes in the number of metabolites in bone and serum with CHC compared to NHC indicated that CHC caused greater metabolic changes. Besides the six discussed above, the influence of other metabolites observed in this metabolomics study awaits further investigation.

Our research has limitations. Firstly, we did not examine rotational forces (torsion or twisting moments) in long bones, which are functionally relevant because these are common type of stress experienced by bones during daily activities such as walking, running, and jumping. Secondly, while both OCs inhibited the ability to mineralize the matrix, the spatial distribution of mineralization in bone by Backscattered electron microscopy, which provides additional insights into the mechanical properties of the bone, was not measured. Thirdly, our study did not consider an important aspect of bone quality that can be determined by Atomic Force Microscopy, where a smooth bone surface may indicate better bone quality with less susceptibility to damage, whereas a rough surface may indicate the opposite. Fourthly, Raman spectroscopy measures the degree of polarization of light scattered by collagen fibers to determine the orientation of collagen that determines bone strength and its resistance to deformation under mechanical stress, and we have not assessed it. Fifthly, although high bone turnover rate measured by serum markers (C-terminal telopeptide, propeptides of type 1 collagen, alkaline phosphatase, and osteocalcin) are associated with an increased risk of fractures in humans, we did not measure them. Instead, we focused on direct measurements of bone strength in rats that goes beyond the fracture risk obtained from the turnover markers. Sixthly, we did not measure estradiol (a form of estrogen). Instead, we measured SHBG which binds estradiol and renders it inactive. As a result, total estrogen levels, which include both bound and free estrogen, may not accurately reflect the bioactive form of estrogen. Studies on older women have shown that SHBG, but not testosterone or estradiol levels, were independently associated with an increased risk of vertebral fractures (43, 44). This finding suggests that measurement of SHBG in the context of bone health in response to OCs is relevant and instructive. Lastly, without the normative values for bone parameters that were changed by the OCs in rats, it is difficult to determine whether the changes are clinically significant or not.

Based on our findings, it appears that after 7 months of treatment, CHC had a more detrimental impact on bone health than NHC. This was demonstrated by a decrease in bone mass independent of body weight, mineralization, and strength, as well as deterioration in bone microarchitecture, and changes in a greater number of metabolites detected in bone and serum. The decrease in bone mass at L5 caused by the OCs is consistent with the effect of CHCs in adolescent women (45), implying that the results of our study on bone mineralization, microarchitecture, composition, and strength in response to these OCs, may apply to humans.

## Data availability statement

The original contributions presented in the study are included in the article/**Supplementary Material**. Further inquiries can be directed to the corresponding author.

## Ethics statement

The animal study was approved by CSIR-CDRI National Laboratory Animal Centre with animal ethics Committee approval no: IAEC/2021/66/Renew-0/Sl no.14 dated 29.06.2021. The study was conducted in accordance with the local legislation and institutional requirements.

## Author contributions

KP: Investigation, data curation, formal analysis, writing - original draft. SSh: data curation, formal analysis. SK: Bone quality data curation, Formal analysis. MT: Metabolomics data curation and Formal analysis. SSa: data curation, formal analysis. SR: data curation, formal analysis. CK: data curation, formal analysis. AS: Supervision, methodology. NK: Supervision, methodology. NC: Supervision, conceptualization, methodology, project administration and writing. All authors contributed to the article and approved the submitted version.

## Supplementary material

Supplementary Figure 1 Effect of OCs on body weight and body composition. **(A)** Body weight and delta body weight. **(B)** Percent change in lean mass and fat mass. **(C)** Serum sex hormone-binding globulin (SHBG) levels. (n > 6 rats/group). Ctrl, control; CHC, combined hormonal contraceptive; NHC, non-hormonal contraceptive.

Supplementary Figure 2 Effect of OCs on bone mass and microarchitecture on tibia metaphysis assessed by µCT. Data are expressed as mean ± SEM (n > 6 rats/group). *p<0.05, **p<0.01 and ***p<0.001 compared with the age-matched control (vehicle treated). Ctrl, control; CHC, combined hormonal contraceptive; NHC, non-hormonal contraceptive.
