# Supplementary figures and images for: Hormonal and non-hormonal oral contraceptives given long-term to pubertal rats differently affect bone mass, quality and metabolism
Source: Front Endocrinol (Lausanne). 2023 Aug 17;14:1233613. doi: 10.3389/fendo.2023.1233613 (PMC10470083; doi:10.3389/fendo.2023.1233613)

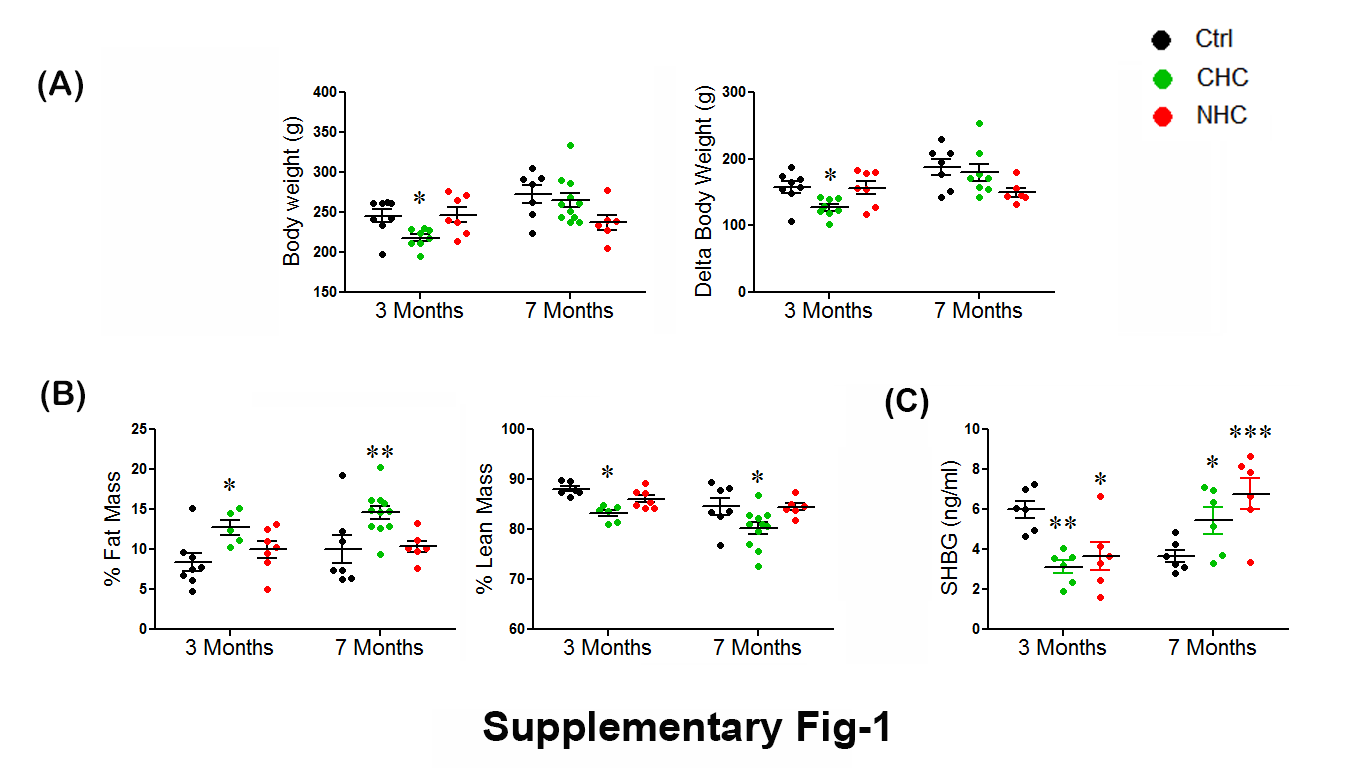

Supplement: Supplementary Figure 1 — Effect of OCs on body weight and body composition. (A) Body weight and delta body weight. (B) Percent change in lean mass and fat mass. (C) Serum sex hormone-binding globulin (SHBG) levels. (n > 6 rats/group). Ctrl, control; CHC, combined hormonal contraceptive; NHC, non-hormonal contraceptive. [file Image_1.tif]

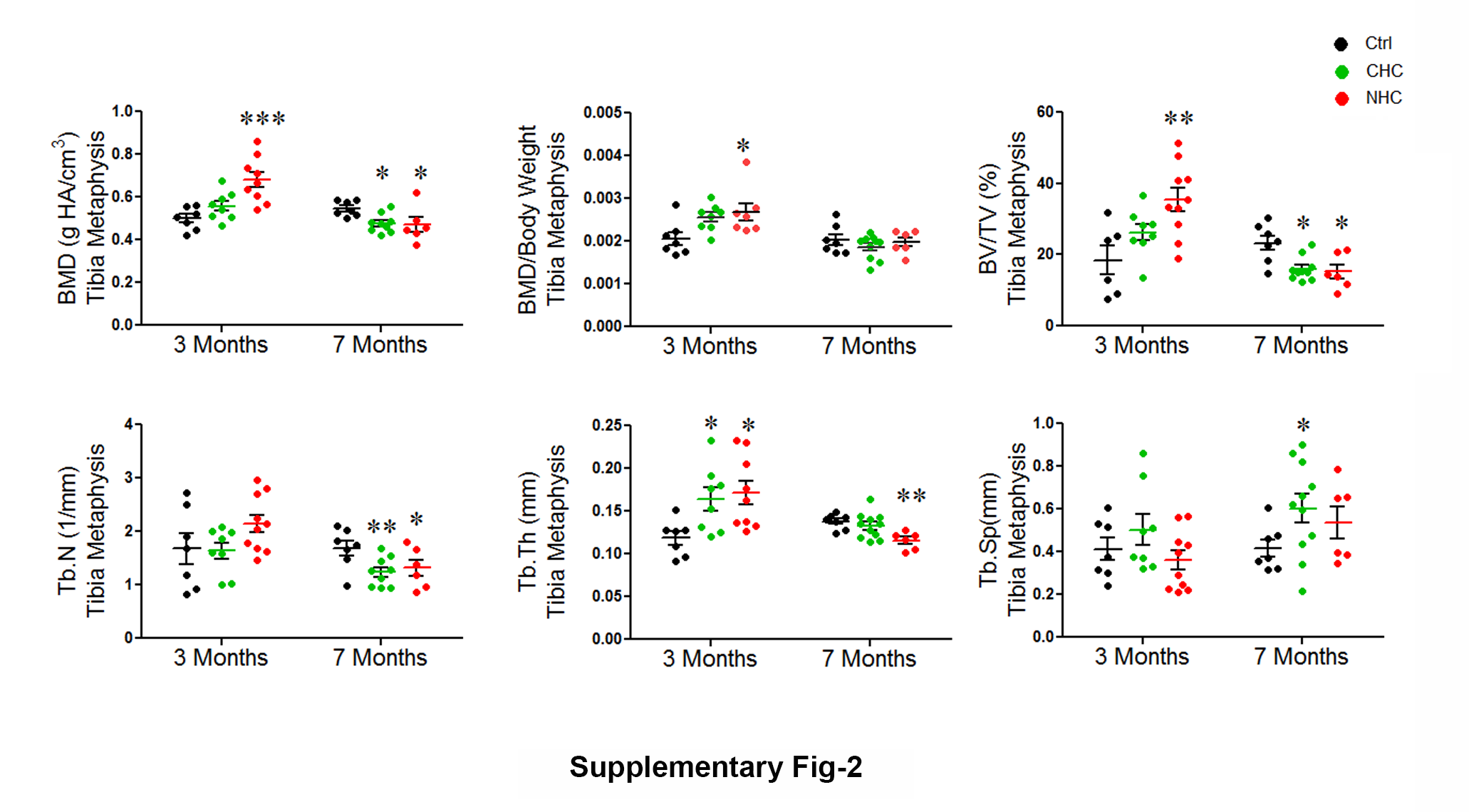

Supplement: Supplementary Figure 2 — Effect of OCs on bone mass and microarchitecture on tibia metaphysis assessed by µCT. Data are expressed as mean ± SEM (n > 6 rats/group). *p<0.05, **p<0.01 and ***p<0.001 compared with the age-matched control (vehicle treated). Ctrl, control; CHC, combined hormonal contraceptive; NHC, non-hormonal contraceptive. [file Image_2.tif]
